# Prognostic value of progesterone receptor expression in ovarian cancer: a meta-analysis

**DOI:** 10.18632/oncotarget.15982

**Published:** 2017-03-07

**Authors:** Hui Luo, Saisai Li, Menghuang Zhao, Bo Sheng, Haiyan Zhu, Xueqiong Zhu

**Affiliations:** ^1^ Department of Obstetrics and Gynecology, The Second Affiliated Hospital of Wenzhou Medical University, Wenzhou, Zhejiang, China

**Keywords:** prognostic, progesterone receptor, ovarian cancer, meta-analysis

## Abstract

**Objective:**

While a prognosis value of progesterone receptor (PR) in ovarian cancer has been reported in some publications, controversial data were presented by different reports. In order to address the disagreement of progesterone receptor in ovarian cancer survival, we conducted this meta-analysis.

**Methods:**

Relevant articles on progesterone receptor and ovarian cancer prognosis were identified via a thorough search of PubMed, Embase and Cochrane Central. Hazard ratios (HR) and 95% confidence interval (CI) were extracted from studies on overall survival (OS) and disease-free survival (DFS)/progress-free survival (PFS)/recurrence-free survival (RFS).

**Result:**

A total of 28 eligible studies containing 5685 patients were collected for analysis. It was found that progesterone receptor positivity was significantly associated with favorable overall survival (OS) (HR = 0.86, 95% CI = 0.78 to 0.95, *P* = 0.002) and disease-free survival (DFS)/progress-free survival (PFS)/recurrence-free survival (RFS) (HR = 0.75, 95% CI = 0.61 to 0.93, *P* = 0.008) of ovarian cancer patients. Subgroup analysis showed that progesterone receptor expression was associated with a favorable prognosis of unclassified ovarian cancer, European origin, and immunohistochemical detection method.

**Conclusion:**

Progesterone receptor expression can be used as a favorable prognostic predictor in ovarian cancer managements.

## INTRODUCTION

Ovarian cancer is the leading cause of death from gynecological malignancy and the fifth cause of death among women worldwide [1]. In 2015, there were approximately 22,280 new cases and 14,240 deaths from ovarian cancer in the United States along [1]. This disease is often late-detected and progresses rapidly, thus has a poor prognosis [2, 3]. Over 70% of the patients are diagnosed at an advanced stage of disease, with a 5-year survival rate of merely 30% [4]. Despite modern management, such as cytoreductive surgery, and subsequent adjuvant chemotherapy, not all patients gain a benefit from these therapies [5, 6]. Moreover, the outcome has revealed that about 60-70% of patients will have recurrent disease within 18 months [7]. Therefore, discovery of applicable prognostic biomarkers for ovarian cancer is urgently required to improve the clinical outcomes of this disease.

Progesterone receptor (PR) is an intracellular polypeptide. Upon binding to progesterone, it translocates into the nucleus, and regulates expression of a specific set of genes [8]. Activation of progestational signaling can suppress ovulation, antagonize the growth-promoting effect of estrogen, and regulate ovarian cancer cell proliferation and apoptosis [9, 10]. Many studies have investigated the relationship between progesterone receptor and ovarian cancer patient outcome [11–13]. However, the correlation between the expression of progesterone receptor and prognosis of ovarian cancer remains controversial [11–38]. For example, Aminah et al. reported that progesterone receptor expression was positively associated with survival and was a favorable prognostic factor in ovarian cancer [14], while Michel et al. showed that progesterone receptor expression had no influence on the survival in ovarian cancer patients [15].

In this study, we performed a meta-analysis of published studies to assess the prognostic value of progesterone receptor in patients with ovarian cancer. The clinical implications of the findings are also discussed.

## RESULTS

### Characteristics of eligible studies

**Figure 1:**
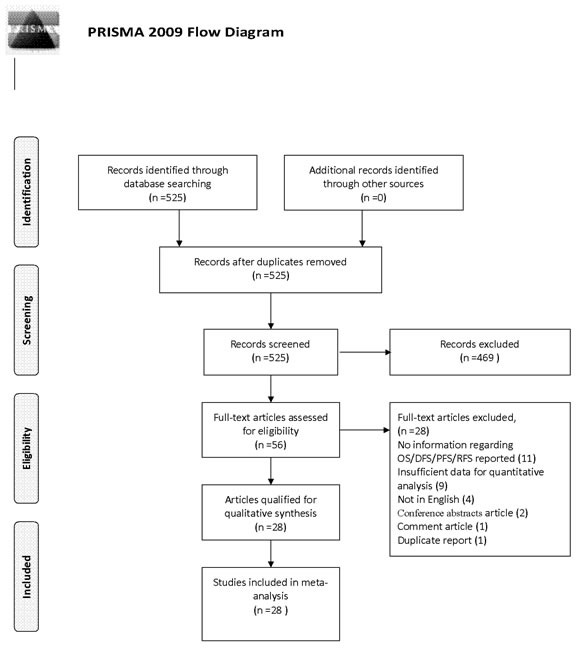
Flow of study identification, inclusion, and exclusion

The primary search yielded a total of 525 citations, among which 469 were excluded after screening of titles, keywords and abstracts. 28 articles were excluded after reviewing the full texts based on the selection criteria (11 studies had no relevant outcomes, 9 studies had no sufficient data, 4 studies were not written in English, 3 articles were conference articles or comments, and one study was duplicated report). Finally, 28 eligible studies involving 5685 patients were enrolled in the meta-analysis (Figure 1).

As for the region, 17 studies were performed in Europe, 6 studies in America, 2 studies in Asia, 2 studies in South America and one study was conducted in mixed populations. The sample-sizes ranged from 40 to 1661, with a median value of 116. A total of 26 studies described the correlation between overall survival (OS) and progesterone receptor expression, while 15 trials involved disease-free survival (DFS)/progress-free survival (PFS)/recurrence-free survival (RFS). Twenty-five studies used immunohistochemical staining for progesterone receptor assessment, one study used ELISA, and the 2 studies used the dextran-coated charcoal method. The quality of included studies was assessed by the Newcastle-Ottawa Scale (NOS), ranging from six to eight scores. Characteristics of detailed features were summarized in Table 1.

**Table 1:** Characteristics of the studies included in this meta-analysis

Study & year	Country	Histological type	Stage	Sample size	Detection method	Age (min-max)	PR (positive/all)	Follow up (months)	Out- comes	HR (95%CI)	Method for data collection	NOS score
Jatoi 2015 [14]	USA	Unclassified	I-IV	490	IHC	NA	193/490	16.8 (median)	RFS	0.64 (0.44-0.94)	Directly	8
Van Kruchten 2015 [15]	Netherland	Unclassified	I-IV	121	IHC	61(30-84) (median)	23/121	45 (median)	OS	1.27 (0.69-2.31)	Directly	8
Van Kruchten 2015 [15]	Netherland	Unclassified	I-IV	121	IHC	61(30-84) (median)	23/121	45 (median)	PFS	0.92 (0.52-1.63)	Directly	8
Jonsson 2015 [11]	Sweden	Unclassified	I-IV	118	IHC	58.4(26-83)(mean)	36/118	60 (all)	OS	0.34 (0.19-0.62)	Directly	8
Jonsson 2015 [11]	Sweden	Unclassified	I-IV	118	IHC	58.4(26-83)(mean)	36/118	60 (all)	PFS	0.42 (0.24-0.71)	Directly	8
Tkalia 2014 [16]	Ukraine	Serous	I-IV	232	IHC	51.7(18-82)(mean)	147/232	39.5±1.7 (mean)	OS	0.98 (0.7-1.39)	Indirectly	7
Tkalia 2014 [16]	Ukraine	Serous	I-IV	232	IHC	51.7(18-82)(mean)	147/232	39.5±1.7 (mean)	RFS	0.89 (0.65-1.2)	Indirectly	7
Matsuo 2014 [17]	USA	Serous	I-IV	112	IHC	62.6±10.6 (mean)	10/112	NA	OS	0.9 (0.36-2.25)	Directly	7
Matsuo 2014 [17]	USA	Serous	I-IV	112	IHC	62.6±10.6 (mean)	10/112	NA	PFS	0.71 (0.31-1.63)	Directly	7
De Toledo 2014 [18]	Brazil	Unclassified	I-IV	152	IHC	55.2±12.3 (mean)	48/152	43.6 (mean)	OS	1.96 (0.91-4.25)	Directly	7
De Toledo 2014 [18]	Brazil	Unclassified	I-IV	152	IHC	55.2±12.3 (mean)	48/152	43.6 (mean)	DFS	1.96 (0.83-4.58)	Directly	7
Battista 2014 [12]	Germany	Unclassified	I-IV	108	IHC	61.7±11.4 (mean)	15/108	43.3(11.4-68) (median)	OS	0.13 (0.03-0.68)	Directly	6
Battista 2014 [12]	Germany	Unclassified	I-IV	108	IHC	61.7±11.4 (mean)	15/108	43.3(11.4-68) (median)	DFS	0.15 (0.03-0.68)	Directly	6
Sieh 2013 [19]	Mix	Serous	I-IV	1661	IHC	60.9 (mean)	124/1661	49.2 (mean)	OS	0.74 (0.58-0.94)	Directly	7
Lenhard 2012 [20]	Germany	Unclassified	I-IV	155	IHC	59(21-88) (median)	108/155	146.4 (median)	OS	0.81 (0.26-2.51)	Indirectly	6
Alonso 2009 [21]	Spain	Unclassified	IIB-IV	62	IHC	56 (median)	40/62	27 (median)	OS	0.98 (0.96-1)	Directly	7
Arias-Pulido 2009 [22]	Mexico	Unclassified	I-IV	134	IHC	54.1(17-87) (median)	64/134	60 (all)	OS	0.38 (0.08-1.89)	Indirectly	7
Athanassiadou 1998 [23]	Greece	Unclassified	NA	100	IHC	51.56±10.2 (mean)	17/100	28.5 (mean)	OS	1.23 (0.74-2.03)	Indirectly	6
Buchynska d 2009[24]	Ukraine	Serous	I-IV	81	IHC	46.6±2.4 (mean)	55/81	60 (all)	OS	0.1 (0.02-0.45)	Indirectly	6
De Sousa Damiao 2007 [25]	Brazil	Unclassified	I-IV	40	IHC	55.8(20-87) (mean)	5/40	120 (all)	OS	1.07 (0.37-3.1)	Directly	8
De Stefano 2011 [26]	Italy	Serous	III-IV	58	IHC	54(33-79) (median)	31/58	35(9-127) (mean)	OS	0.6 (0.3-1.4)	Directly	7
De Stefano 2011 [26]	Italy	Serous	III-IV	58	IHC	54(33-79) (median)	31/58	35(9-127) (mean)	DFS	0.3 (0.1-0.6)	Directly	7
Garcia-Velasco 2009 [27]	Spain	Unclassified	NA	72	IHC	57 (median)	36/72	33 (median)	OS	1.43 (0.47-4.32)	Directly	7
Garcia-Velasco 2009 [27]	Spain	Unclassified	NA	72	IHC	57 (median)	36/72	33 (median)	PFS	1.44 (0.75-2.75)	Directly	7
Hempling 1998 [28]	USA	Unclassified	III-IV	67	IHC	60.1 (mean)	31/67	63.6 (mean)	PFS	0.58 (0.34-0.99)	Indirectly	7
Hornung 2004 [29]	Switzerland	Unclassified	I-IV	111	ELISA	58(21-94) (median)	34/111	87 (all)	OS	0.62 (0.34-1.14)	Indirectly	7
Hornung 2004 [29]	Switzerland	Unclassified	I-IV	111	ELISA	58(21-94) (median)	34/111	87 (all)	DFS	0.79 (0.45-1.42)	Indirectly	7
Hogdall 2007 [30]	Denmark	Unclassified	I-IV	580	IHC	NA	116/580	120 (all)	OS	0.69 (0.51-0.94)	Directly	7
Lee 2005 [31]	USA	Unclassified	I-IV	322	IHC	58.3(20-86) (mean)	278/322	64(1-120) (mean)	OS	1.6 (1.1-2.4)	Directly	7
Liu 2009 [32]	USA	Serous	III-IV	148	IHC	NA	57/131	100 (all)	OS	0.97 (0.61-1.55)	Indirectly	8
Liu 2010 [33]	China	Unclassified	I-IV	116	IHC	49(30-76) (median)	62/116	43(5-93) (median)	OS	1.2 (0.67-2.16)	Indirectly	7
Liu 2010 [33]	China	Unclassified	I-IV	116	IHC	49(30-76) (median)	62/116	43(5-93) (median)	PFS	0.81 (0.47-1.41)	Indirectly	7
Scambia 1995 [29]	Italy	Unclassified	I-IV	117	DCC	NA	40/113	19(2-110) (median)	OS	1.13 (0.59-2.14)	Indirectly	7
Scambia 1995 [34]	Italy	Unclassified	I-IV	117	DCC	NA	40/113	19(2-110) (median)	PFS	1.04 (0.6-1.79)	Indirectly	7
Schlumbrecht 2011 [35]	USA	Serous	III-IV	83	IHC	62.6 (34.5-85.9) (mean)	NA	38.7(0.5-67.8) (median)	OS	0.99 (0.95-1.02)	Directly	7
Schlumbrecht 2011 [35]	USA	Serous	III-IV	83	IHC	62.6 (34.5-85.9) (mean)	NA	38.7(0.5-67.8) (median)	RFS	0.99 (0.99-1.01)	Directly	7
Sinn 2011 [13]	Germany	Unclassified	I-IV	143	IHC	NA	45/143	220 (all)	OS	0.36 (0.15-0.82)	Indirectly	7
Sinn 2011 [13]	Germany	Unclassified	I-IV	143	IHC	NA	45/143	220 (all)	PFS	0.51 (0.34-0.77)	Indirectly	7
Slotman 1990 [36]	Netherlands	Unclassified	I-IV	100	DCC	60.2(17-86) (mean)	53/100	64.8(48-78) (mean)	OS	0.52 (0.28-0.96)	Indirectly	7
Tomsova 2008 [37]	Czech Republic	Unclassified	I-IV	116	IHC	53(27-82) (median)	NA	39(1-120) (median)	OS	0.4 (0.22-0.7)	Directly	7
Yang 2009 [38]	China	Unclassified	I-IV	86	IHC	34.2(17-40) (median)	49/86	120 (all)	OS	0.52 (0.32-0.69)	Directly	7

Abbreviations: HR: hazard ratio, CI: confidence interval, IHC: immunohistochemistry, ELISA: enzyme-linked immunosorbent assay, DCC: dextran-coated charcoal, NA: not available, OS: overall survival, DFS/PFS/RFS: disease-free survival/ progress-free survival/ recurrence-free survival, Serous: serous ovarian cancer, Unclassified: serous, mucinous, clear cell, endometrioid, transitional cell, undifferentiated, differentiated, and others.

### Association of progesterone receptor expression with OS of ovarian cancer

**Figure 2:**
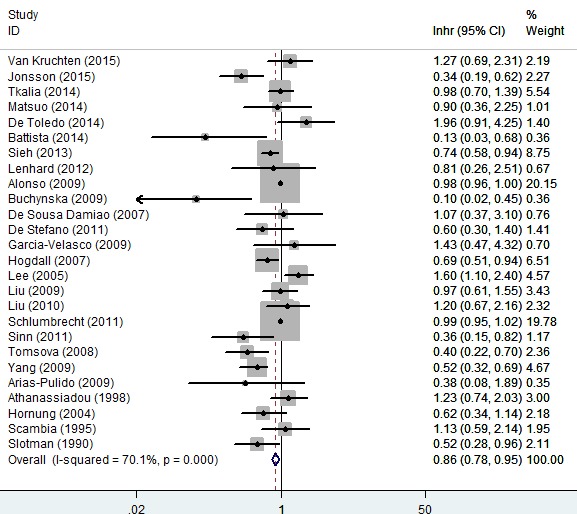
Forest plot of HR and 95% CI of the association between progesterone receptor expression and overall survival of ovarian cancer Summary of all 26 trails, the results showed progesterone receptor was associated with a favorable OS of ovarian cancer using random effects model. The % weight was computed automatically by the Stata software.

**Figure 3:**
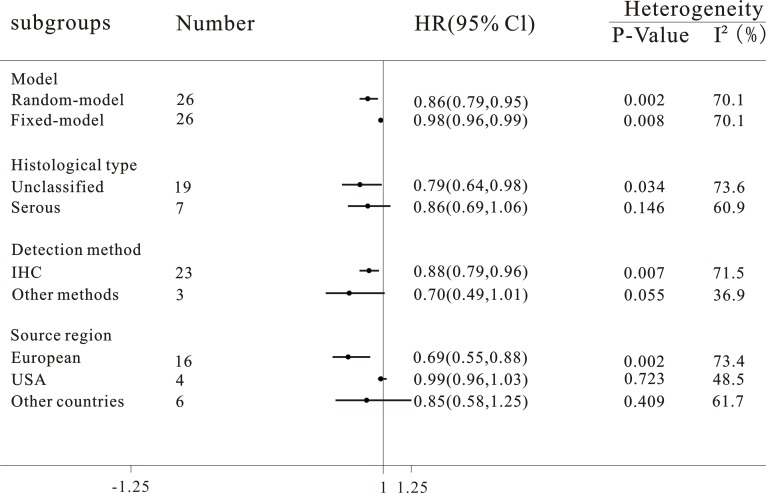
Subgroup analyses of the relationship between progesterone receptor expression and overall survival of ovarian cancer

The results of progesterone receptor expression with OS were listed in Figure 2. The combined analysis of 26 studies showed that the expression of progesterone receptor was associated with a favorable OS of ovarian cancer (HR = 0.86, 95% CI = 0.78 to 0.95, *P* = 0.002). Significant heterogeneity was shown among these studies (*I*^2^ = 70.1%). Thus a random-effects model was used for statistical analysis, and subgroup meta-analysis was performed to investigate the possible source of the heterogeneity among these studies (Figure 3).

In the subgroup analysis by histological type, a better OS was strongly linked to progesterone receptor expression in unclassified ovarian cancer (*n* = 19, HR = 0.79, 95% CI = 0.64 to 0.98, *P* = 0.034, *I*^2^ = 73.6%) while there was no significant association in serous ovarian cancer (*n* = 7, HR = 0.85, 95% CI = 0.69 to 1.06, *P* = 0.146, *I*^2^ = 60.9%).

With regard to different detection methods of progesterone receptor in ovarian cancer, it was found that progesterone receptor expression was associated with a favorable OS of ovarian cancer in immunohistochemical staining group (*n* = 23, HR = 0.88, 95% CI = 0.79 to 0.96, *P* = 0.007, *I*^2^ = 71.5%). Nevertheless, the expression of progesterone receptor was irrelevant with OS of ovarian cancer patients by using other detection methods (*n* = 3, HR = 0.70, 95% CI = 0.49 to 1.01, *P* = 0.055, I^2^ = 36.9%).

There were three stratified subgroups about source regions of included studies, a pooled HR was 0.69 (*n* = 16, 95% CI = 0.55 to 0.88, *P* = 0.002, *I*^2^ = 73.4%) in European population, indicating that progesterone receptor positivity exerted favorable influence on OS among these ovarian cancer patients. However, progesterone receptor expression was not associated with OS in American (*n* = 4, HR = 0.99, 95% CI = 0.96 to 1.03, *P* = 0.723, *I*^2^ = 48.5%) and other countries patients (*n* = 6, HR = 0.85, 95% CI = 0.58 to 1.25, *P* = 0.409, *I*^2^ = 61.7%).

### Association of progesterone receptor expression with DFS/PFS/RFS of ovarian cancer

**Figure 4:**
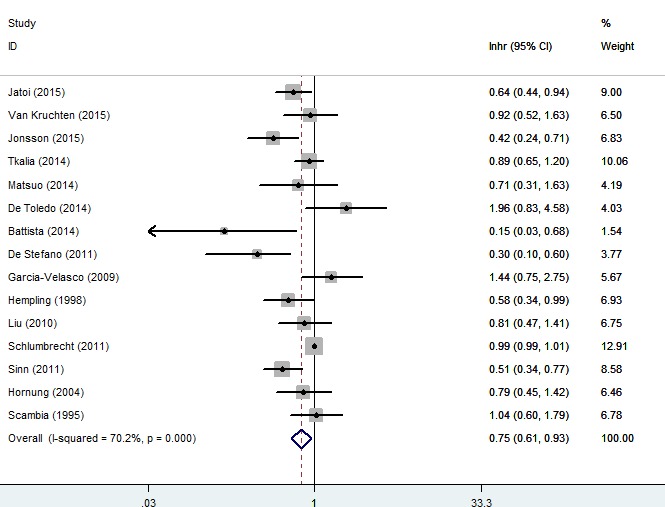
Forest plot of HR and 95% CI of the association between progesterone receptor expression and disease-free survival/progress-free survival/recurrence-free survival of ovarian cancer patients Summary of all 15 trails, the results showed progesterone receptor was associated with a favorable DFS/PFS/RFS of ovarian cancer using random effects model.

**Figure 5:**
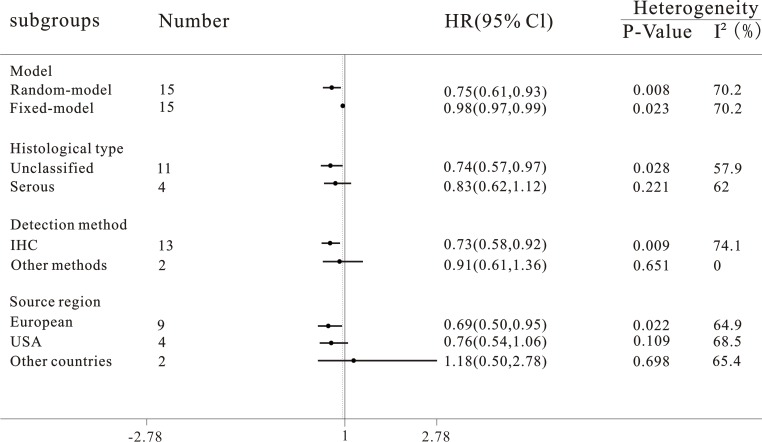
Subgroup analyses of the relationships between progesterone receptor and disease-free survival/progress-free survival/recurrence-free survival of ovarian cancer

We analyzed the relationship between the expression of progesterone receptor and DFS/PFS/RFS among ovarian cancer patients. As shown in Figure 4, pooling analysis suggested that expression levels of progesterone receptor predicted an improved DFS/PFS/RFS, both in random (HR = 0.75, 95% CI = 0.61 to 0.93, *P* = 0.008) and fixed model (HR = 0.98, 95% CI = 0.97 to 0.99, *P* = 0.023), along with a moderate heterogeneity of the data (*I*^2^ = 70.2%). Random model was used to calculate the final outcome (Figure 4). Moreover, subgroup meta-analysis was used to stratify the data from selected articles to explore the possible source of the heterogeneity (Figure 5).

In the stratified analysis by histological types of cancers, progesterone receptor expression was associated with a better DFS/PFS/RFS of unclassified ovarian cancer (*n* = 11, HR = 0.74, 95% CI = 0.57 to 0.97, *P* = 0.028, *I*^2^ = 57.9%), while no significant association between progesterone receptor expression and DFS/PFS/RFS was detected in serous ovarian cancer patients (*n* = 4, HR = 0.83, 95% CI = 0.62 to 1.12, *P* = 0.221, *I*^2^ = 62 %).

Divided by detection approaches among the subgroups, immunohistochemical staining group showed a beneficial effect on DFS/PFS/RFS outcome, along with a significant heterogeneity observed (*n* = 13, HR = 0.73, 95% CI = 0.58 to 0.92, *P* = 0.009, *I*^2^ = 74.1%). However, progesterone receptor was not identified as a DFS/PFS/RFS prognostic predictor (*n* = 2, HR = 0.91, 95% CI = 0.61 to 1.36, *P* = 0.651) in ovarian cancer when other detection methods were used.

The included studies were stratified into the European group, American group and the group of other countries. Elevated progesterone receptor expression was found to be positively correlated with DFS/PFS/RFS among European populations (*n* = 9, HR = 0.69, 95% CI = 0.5 to 0.95, *P* = 0.022, *I*^2^ = 64.9%), but not in the American (n = 4, HR = 0.76, 95% CI = 0.54 to 1.06, *P* = 0.109, I^2^ = 68.5%) and the other country populations (*n* = 2, HR = 1.18, 95% CI = 0.5 to 2.78, *P* = 0.698, *I*^2^ = 65.4%).

### Sensitivity analysis

**Figure 6:**
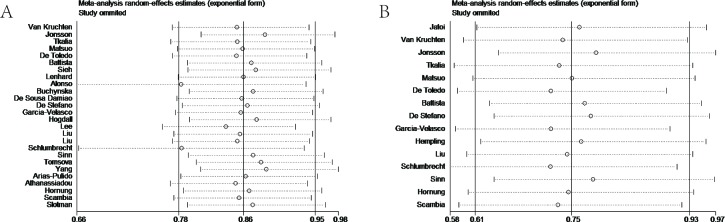
**A.** Sensitivity analysis of the association between progesterone receptor expression and overall survival in ovarian cancer patients. **B.** Sensitivity analysis of the association between progesterone receptor expression and disease-free survival/progress-free survival/recurrence-free survival in ovarian cancer patients. The leave-one-out method was used to confirm the stability of the results.

In sensitivity analysis, the leave-one-out method was chosen to confirm the stability of the results. Eligible studies were sequentially excluded one by one to evaluate the stability of the obtained conclusions from remaining data. After leaving out any single study, statistical significance of the OS or DFS/PFS/RFS was not altered (Figure 6A, 6B).

### Publication bias

**Figure 7:**
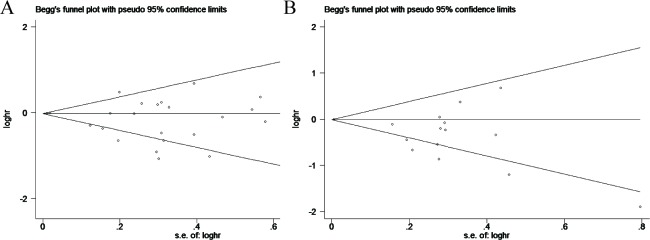
**A.** Funnel plot for the publication bias test between progesterone receptor expression and overall survival. **B.** Funnel plot for the publication bias test between progesterone receptor expression and disease-free survival/progress-free survival/recurrence-free survival. Visual inspection of the Begg's funnel plot did not indicated substantial asymmetry.

**Figure 8:**
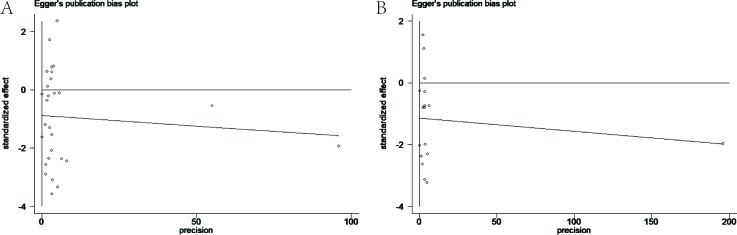
**A.** Egger's publication bias plot of the studies assessing progesterone receptor and overall survival in ovarian cancer. **B.** Egger's publication bias plot of the studies assessing progesterone receptor expression and disease-free survival/progress-free survival/recurrence-free survival of ovarian cancer patients. Visual inspection of the Egger's funnel plot did not indicate substantial asymmetry.

Funnel plots (Figure 7) analyses were graphically symmetric, and Begg's test (*P* = 0.481 for OS; *P* = 0.921 for DFS/PFS/RFS) revealed that there was no publication bias among the included studies (Figure 7A, 7B). The Egger's publication bias plot presented no proof of obvious publication bias (*P* = 0.467 for OS; *P* = 0.882 for DFS/PFS/RFS), suggesting stable conclusions (Figure 8A, 8B).

## DISCUSSION

In this study, we systematically evaluated the prognostic value of progesterone receptor expression in 5685 ovarian cancer patients from 28 different studies and demonstrated that the expression of progesterone receptor was an indicator of a favorable prognosis for ovarian cancer patients. Progesterone receptor has been widely described to be associated with ovarian cancer. A marked downregulation of progesterone receptor mRNA expression was noted in ovarian cancer cell lines when compared with normal ovarian surface epithelium cells [39]. Moreover, progesterone receptor immunopositivity was observed in the majority of borderline tumors, whereas a large percentage (93%) of malignant ovarian tumors stained negative for progesterone receptor [40]. Thus, loss of progesterone receptor maybe involved in the etiology and progression of ovarian cancer. Multiple *in vitro* studies have shown that an increased progesterone receptor expression could promote the progesterone-induced inhibition of DNA synthesis, cell division, proliferation and apoptosis in ovarian cancer cells [8, 41]. Such effects may partially explain the positive correlation between progesterone receptor expression and longer ovarian cancer patient survival.

Ovarian cancers consist of many histological subtypes, including those of serous, mucinous, endometrioid, and clear cell cancers. Among the selected publications, only 7 studies concentrated on serous ovarian cancer while the remaining 21 studies involved all subtypes of ovarian cancers. Progesterone receptor expression was linked to a better OS and DFS/PFS/RFS in unclassified ovarian cancers, while progesterone receptor expression was related to neither OS nor DFS/PFS/RFS in serous type of cancers. Therefore, we suggest that the expression of progesterone receptor may be a prognostic biomarker in non-serous ovarian cancer rather than serous ovarian cancer. It is recognized that prognostic markers varied substantially across subtype in ovarian cancers [42]. Progesterone receptor expression could be related to certain biological characteristics such as response to treatment [19].

Subgroup analyses indicated the presence of remarkable influence of progesterone receptor on OS and DFS/PFS/RFS of ovarian cancer when using immunohistochemical technology, while this positive prognostic value was not observed with the use of other detection methods. Immunohistochemical technology has been widely used to identify the location and expression of proteins in patients’ tissues. It is possible that the information of progesterone receptor localization in cancers provided by this technique afford a specific diagnostic power over other techniques. Given only three studies used other methods, an alternative possibility could be that the differences were caused by the small sample size. Further studies are needed to clarify the prognostic value of progesterone receptor detected by other methods.

With respect to source regions, the expression of progesterone receptor was tightly related to a better prognosis in European population, while progesterone receptor expression had no effect on the prognosis in American group and other country group. It has been noted that several genes exert different effects on cancer risk and prognosis across ethnic groups. For example, patients with high expression of C-X-C chemokine receptor 4 (CXCR4) have a poor prognosis among Asian women with ovarian cancer. However, this relationship was not observed among ovarian cancer patients from non-Asian regions [43]. A probable explanation for such divergence is that genetic background, life style and environmental effect varied among different ethnic groups/regions. These differences may modify the specific correlation between progesterone expression and ovarian cancer outcomes.

There are several important implications in this meta-analysis. First, the study shows that the expression of progesterone receptor is related to a favorable outcome of ovarian cancer, suggesting progesterone receptor may be a potential prognostic marker for ovarian cancers. Second, the study conducted subgroup analyses based on histological type, detection method and source region to systematically evaluate the prognostic effect on ovarian cancer patients. Finally, the statistical results of the analyses appear to be reliable since both the random-model and fixed-model analyses showed similar results.

This quantitative meta-analysis had its limitations. First, several HRs and 95% CIs were obtained based on the survival curves, which might cause bias to the result. Second, despite the usage of subgroup analysis, the heterogeneity across studies could not to be eliminated completely, which could result in bias of the outcome. Finally, we performed the sub-group analysis only based on progesterone receptor expression and ovarian cancer with OS or DFS/PFS/RFS, due to the lack of effective data. Therefore, further investigations are needed to address the above-mentioned shortcomings.

In summary, this meta-analysis shows that progesterone receptor positivity is associated with an improved OS and DFS/PFS/RFS, and progesterone receptor expression could be an indicator of a favorable prognosis in ovarian cancer patients, especially when measured by immunohistochemistry.

## MATERIALS AND METHODS

### Search strategy

In this Meta-analysis, we performed a comprehensive search for available literatures in electronic databases of PubMed, Embase and Cochrane Central until August 31, 2016. The following search terms were used to identify studies: (PR or “progesterone receptor”) and (“ovarian neoplasm” or “ovarian cancer” or “ovarian carcinoma”) and (prognosis or outcome or survival). In order to guarantee the accuracy and completeness, both abstracts and full texts were screened in detail to exclude irrelevant articles.

### Selection criteria

To be eligible, studies must meet the following inclusion criteria: (1) Studies covered the correlation between progesterone receptor expression and clinical prognosis among ovarian cancer patients; (2) Studies provided adequate data for extracting and calculating individual HRs and 95% CIs; (3) Original article was written in English.

Studies were excluded due to the following reasons: (1) Duplicated or overlapped studies; (2) Review articles or case reports or conference papers; (3) Articles were not related to ovarian cancer or progesterone receptor.

Two investigators (Hui Luo and Haiyan Zhu) carried out this procedure independently and any disagreement was resolved by consensus.

### Data extraction

According to predefined standardized extraction forms, data from each qualified study were extracted by two investigators independently. The collected data included the first author, year of publication, country of origin, histological type, tumor stage, total number of patients, detection method, age, progesterone receptor expression status, follow-up time, outcome endpoint, univariate or multivariate hazard ratio (HR) and the 95% confidence interval (95% CI) for progesterone receptor positive-expression group versus progesterone receptor negative-expression group. Survival data (HR with 95% CI) were extracted from tables or texts of included studies. If the definite information of the statistical variables was not provided in an article, sending an email to the author for the original data was the first choice. If the articles had Kaplan-Meier curves, Get Data Graph Digitizer 2.2 was used to digitize and extract survival information [44]. Multivariate HR and 95% CI were selected when both univariate and multivariate results were reported in an individual study.

### Methodological assessment

As this meta analysis was based on observational studies, a Newcastle-Ottawa Scale (NOS) was adopted for the methodological evaluation. Studies graded with more than five scores were classified as high quality trials in methodology [45].

### Statistical analysis

Data were extracted from the primary publications and meta-analysis was conducted with the use of Stata 12.0 analysis software (Stata Corp LP, College Station, TX). Outcome endpoints were divided into two groups, OS and DFS/PFS/RFS, based on the data acquired.

To investigate the source of heterogeneity, we performed the χ2-based Q-test and I^2^ test. If *P* < 0.05 or I^2^ > 50%, which indicating significant heterogeneity among included studies, a random effect model was used to calculate the pooled HR. Otherwise fixed effect model was used. Additionally, a sensitivity analysis examining the consistency of the pooled outcomes was applied. The internal publication bias across the included studies was statistically investigated via Funnel plots as well as Begg's test and Egger's test. All the statistical tests were two-sided, and statistical significance was signified as *P* less than 0.05.

## References

[1] Siegel RL, Miller KD, Jemal A (2016). Cancer statistics, 2016. CA Cancer J Clin.

[2] Chudecka-Głaz AM (2015). ROMA, an algorithm for ovarian cancer. Clin Chim Acta.

[3] Chang SJ, Bristow RE, Ryu HS (2012). Impact of complete cytoreduction leaving no gross residual disease associated with radical cytoreductive surgical procedures on survival in advanced ovarian cancer. Ann Surg Oncol.

[4] Kipps E, Tan DS, Kaye SB (2013). Meeting the challenge of ascites in ovarian cancer: new avenues for therapy and research. Nat Rev Cancer.

[5] Banerjee S, Kaye SB (2013). New strategies in the treatment of ovarian cancer: current clinical perspectives and future potential. Clin Cancer Res.

[6] Liu J, Matulonis UA (2014). New strategies in ovarian cancer: translating the molecular complexity of ovarian cancer into treatment advances. Clin Cancer Res.

[7] Davidson B, Trope CG (2014). Ovarian cancer: diagnostic, biological and prognostic aspects. Womens Health (Lond).

[8] Modugno F, Laskey R, Smith AL, Andersen CL, Haluska P, Oesterreich S (2012). Hormone response in ovarian cancer: time to reconsider as a clinical target?. Endocr Relat Cancer.

[9] Modugno F, Laskey R, Smith AL, Andersen CL, Haluska P, Oesterreich S (2012). Hormone response in ovarian cancer: time to reconsider as a clinical target?. Endocr Relat Cancer.

[10] Shayanfar N, Mashayekh M, Mohammadpour M (2010). Expression of progesterone receptor and proliferative marker ki-67 in various grades of meningioma. Acta Med Iran.

[11] Jonsson JM, Skovbjerg Arildsen N, Malander S, Masback A, Hartman L, Nilbert M, Hedenfalk I (2015). Sex steroid hormone receptor expression affects ovarian cancer survival. Transl Oncol.

[12] Battista MJ, Mantai N, Sicking I, Cotarelo C, Weyer V, Lebrecht A, Solbach C, Schmidt M (2014). Ki-67 as an independent prognostic factor in an unselected cohort of patients with ovarian cancer: Results of an explorative, retrospective study. Oncol Rep.

[13] Sinn BV, Darb-Esfahani S, Wirtz RM, Budczies J, Sehouli J, Chekerov R, Dietel M, Denkert C (2011). Evaluation of a hormone receptor-positive ovarian carcinoma subtype with a favourable prognosis by determination of progesterone receptor and oestrogen receptor 1 mRNA expression in formalin-fixed paraffin-embedded tissue. Histopathology.

[14] Jatoi A, Vierkant RA, Hawthorne KM, Budczies J, Sehouli J, Chekerov R, Dietel M, Denkert C (2016). Clinical and emergent biomarkers and their relationship to the prognosis of ovarian cancer. Oncology.

[15] Van Kruchten M, Van der Marel P, De Munck L, Hollema H, Arts H, Timmer-Bosscha H, de Vries E, Hospers G, Reyners A (2015). Hormone receptors as a marker of poor survival in epithelial ovarian cancer. Gynecol Oncol.

[16] Tkalia IG, Vorobyova LI, Svintsitsky VS, Nespryadko SV, Goncharuk IV, Lukyanova NY, Chekhun VF (2014). Clinical significance of hormonal receptor status of malignant ovarian tumors. Exp Oncol.

[17] Matsuo K, Sheridan TB, Mabuchi S, Yoshino K, Hasegawa K, Studeman KD, Im DD, Rosenshein NB, Roman LD, Sood AK (2014). Estrogen receptor expression and increased risk of lymphovascular space invasion in high-grade serous ovarian carcinoma. Gynecol Oncol.

[18] De Toledo MC, Sarian LO, Sallum LF, Andrade LL, Vassallo J, de Paiva Silva GR, Pinto GA, Soares FA, Fonseca CD, Derchain SF (2014). Analysis of the contribution of immunologically-detectable HER2,steroid receptors and of the “triple-negative” tumor status to disease-free and overall survival of women with epithelial ovarian cancer. Acta Histochem.

[19] Sieh W, Kobel M, Longacre TA, Bowtell DD, deFazio A, Goodman MT, Hogdall E, Deen S, Wentzensen N, Moysich KB, Brenton JD, Clarke BA, Menon U (2013). Hormone-receptor expression and ovarian cancer survival: an Ovarian Tumor Tissue Analysis consortium study. Lancet Oncol.

[20] Lenhard M, Tereza L, Heublein S, Ditsch N, Himsl I, Mayr D, Friese K, Jeschke U (2012). Steroid hormone receptor expression in ovarian cancer: progesterone receptor B as prognostic marker for patient survival. BMC Cancer.

[21] Alonso L, Gallego E, González FJ, Sanchez-Munoz A, Torres E, Pajares BI, Leeflang S, Baha C (2009). Gonadotropin and steroid receptors as prognostic factors in advanced ovarian cancer: a retrospective study. Clin Transl Oncol.

[22] Arias-Pulido H, Smith HO, Joste NE, Bocklage T, Qualls CR, Chavez A, Prossnitz ER, Verschraegen CF (2009). Estrogen and progesterone receptor status and outcome in epithelial ovarian cancers and low malignant potential tumors. Gynecol Oncol.

[23] Athanassiadou P, Sakellariou V, Petrakakou E, Athanassiades P, Zerva C, Liossi A, Michalas S (1998). Cathepsin D immunoreactivity in ovarian cancer correlation with prognostic factors. Pathol Oncol Res.

[24] Buchynska LG, Iurchenko NP, Grinkevych VM, Nesina IP, Chekhun SV, Svintsitsky VS (2009). Expression of the estrogen and progesterone receptors as prognostic factor in serous ovarian cancers. Exp Oncol.

[25] De Sousa Damiao R, Fujiyama Oshima CT, Stavale JN, Gonçalves WJ, Stavale JN (2007). Analysis of the expression of estrogen receptor, progesterone receptor and chicken ovalbumin upstream promoter-transcription factor I in ovarian epithelial cancers and normal ovaries. Oncol Rep.

[26] De Stefano I, Zannoni GF, Prisco MG, Fagotti A, Tortorella L, Vizzielli G, Mencaglia L, Scambia G, Gallo D (2011). Cytoplasmic expression of estrogen receptor beta (ERβ) predicts poor clinical outcome in advanced serous ovarian cancer. Gynecol Oncol.

[27] García-Velasco A, Mendiola C, Sanchez-Munoz A, Ballestín C, Colomer R, Cortés-Funes H (2008). Prognostic value of hormonal receptors, p53, ki67 and HER2/neu expression in epithelial ovarian carcinoma. Clin Transl Oncol.

[28] Hempling RE, Piver MS, Eltabbakh GH, Recio FO (1998). Progesterone receptor status is a significant prognostic variable of progression-free survival in advanced epithelial ovarian cancer. Am J Clin Oncol.

[29] Hornung R, Urs E, Serenella E, Edward W, Ursula S, Urs H, Daniel F (2004). Analysis of potential prognostic factors in 111 patients with ovarian cancer. Cancer Lett.

[30] Hogdall EV, Christensen L, Hogdall CK, Blaakaer J, Gayther S, Jacobs IJ, Christensen IJ, Kjaer SK (2007). Prognostic value of estrogen receptor and progesterone receptor tumor expression in Danish ovarian cancer patients from the “MALOVA” ovarian cancer study. Oncol Rep.

[31] Lee P, Rosen DG, Zhu C (2005). Expression of progesterone receptor is a favorable prognostic marker in ovarian cancer. Gynecol Oncol.

[32] Liu JF, Hirsch MS, Lee H, Matulonis UA (2009). Prognosis and hormone receptor status in older and younger patients with advanced-stage papillary serous ovarian carcinoma. Gynecol Oncol.

[33] Liu N, Wang X, Sheng X (2010). The clinicopathological characteristics of ‘triple-negative’ epithelial ovarian cancer. J Clin Pathol.

[34] Scambia G, Benedetti-Panici P, Ferrandina G, Distefano M, Salerno G, Romanini ME, Fagotti A, Mancuso S (1995). Epidermal growth factor, oestrogen and progesterone receptor expression in primary ovarian cancer: correlation with clinical outcome and response to chemotherapy. Br J Cancer.

[35] Schlumbrecht MP, Xie SS, Shipley GL, Urbauer DL, Broaddus RR (2011). Molecular clustering based on ERαand EIG121 predicts survival in high-grade serous carcinoma of the ovary/peritoneum. Mod Pathol.

[36] Slotman BJ, Nauta JJ, Rao BR (1990). Survival of patients with ovarian cancer apart from stage and grade, tumor progesterone receptor content is a prognostic indicator. Cancer.

[37] Tomsova M, Melichar B, Sedlakova I, Steiner I (2008). Prognostic significance of CD3+ tumor-infiltrating lymphocytes in ovarian carcinoma. Gynecol Oncol.

[38] Yang XY, Xi MR, Yang KX, Yu H (2009). Prognostic value of estrogen receptor and progesterone receptor status in young Chinese ovarian carcinoma patients. Gynecol Oncol.

[39] Lau KM, Mok SC, Ho SM (1999). Expression of human estrogen receptor-alpha and -beta, progesterone receptor, and androgen receptor mRNA in normal and malignant ovarian epithelial cells. Proc Natl Acad Sci U S A.

[40] Noguchi T1, Kitawaki J, Tamura T, Kim T, Kanno H, Yamamoto T, Okada H (1993). Relationship between aromatase activity and steroid receptor levels in ovarian tumors from postmenopausal women. J Steroid Biochem Mol Biol.

[41] Lindgren P, Backstrom T, Mahlck CG, Ridderheim M, Cajander S (2001). Steroid receptors and hormones in relation to cell proliferation and apoptosis in poorly differentiated epithelial ovarian tumors. Int J Oncol.

[42] Zhu H, Zhu X, Zheng L, Hu X, Sun L, Zhu X (2016). The role of the androgen receptor in ovarian cancer carcinogenesis and its clinical implications. Oncotarget.

[43] Liu CF, Liu SY, Min XY, Ji YY, Wang N, Liu D, Ma N, Li ZF, Li K (2014). The prognostic value of CXCR4 in ovarian cancer: a meta-analysis. PLoS One.

[44] Cheng J, Gao J, Tao K, Yu P (2016). Prognostic role of Gli1 expression in solid malignancies: a meta-analysis. Sci Rep.

[45] Zhu H, Luo H, Zhu X, Hu X, Zheng L, Zhu X (2017). Pyruvate kinase M2 (PKM2) expression correlates with prognosis in solid cancers: a meta-analysis. Oncotarget.

